# Monitoring glycosylation metabolism in brain and breast cancer by Raman imaging

**DOI:** 10.1038/s41598-018-36622-7

**Published:** 2019-01-17

**Authors:** M. Kopec, A. Imiela, H. Abramczyk

**Affiliations:** Lodz University of Technology, Faculty of Chemistry, Institute of Applied Radiation Chemistry, Laboratory of Laser Molecular Spectroscopy, Wroblewskiego 15, 93-590 Lodz, Poland

## Abstract

We have shown that Raman microspectroscopy is a powerful method for visualization of glycocalyx offering cellular interrogation without staining, unprecedented spatial and spectral resolution, and biochemical information. We showed for the first time that Raman imaging can be used to distinguish successfully between glycosylated and nonglycosylated proteins in normal and cancer tissue. Thousands of protein, lipid and glycan species exist in cells and tissues and their metabolism is monitored via numerous pathways, networks and methods. The metabolism can change in response to cellular environment alterations, such as development of a disease. Measuring such alterations and understanding the pathways involved are crucial to fully understand cellular metabolism in cancer development. In this paper Raman markers of glycogen, glycosaminoglycan, chondroitin sulfate, heparan sulfate proteoglycan were identified based on their vibrational signatures. High spatial resolution of Raman imaging combined with chemometrics allows separation of individual species from many chemical components present in each cell. We have found that metabolism of proteins, lipids and glycans is markedly deregulated in breast (adenocarcinoma) and brain (medulloblastoma) tumors. We have identified two glycoforms in the normal breast tissue and the malignant brain tissue in contrast to the breast cancer tissue where only one glycoform has been identified.

## Introduction

The most important components of cells are proteins, lipids and carbohydrates. Proteins have specific functions, such as catalyzing chemical reactions, facilitating communication between different cells, or transporting biological molecules. Lipids and carbohydrates are used primarily as source of energy, but it has been shown recently that they move along intersecting sets of metabolic pathways with impact on human diseases, such as cancer^1–5^.

Knowledge about glycans, sequences of carbohydrates conjugated to proteins and lipids, lag behind mainstream fields of proteomics and lipidomics. Recent years have begun to accelerate research on glycans, the most abundant and structurally diverse type of posttranslational modification. Recent advances in glycomics reveal the scope and scale of their functional roles and their impact on human disease^2,3^. Recent acceleration in research on glycans is related to the development of clinical cancer diagnostic markers based on glycoproteins, given the long known alterations in glycans associated with cancer^4–7^.

A great number of clinicopathological studies have shown an evident correlation between aberrant glycosylation status of primary, invasive and metastatic human cancers^8–11^. Glycosylation is a form of co-translational and post-translational modification. The products of glycosylation are involved in a number of processes relevant to carcinogenesis, including regulation of growth factors/growth factor receptors, cell–cell adhesion and motility as well as immune system modulation^11^ and diseases of nervous system^12^.

Glycosylation refers to the enzyme-directed site-specific reaction in which a carbohydrate acting as a glycosyl donor is attached to a hydroxyl or other functional group of another molecule (a glycosyl acceptor such as proteins, lipids or other organic molecules).

Glycosaminoglycans (GAGs) are important subgroup of glycans, because they are major components of the extracellular matrix in human tissues, and play important roles in various physiological processes, such as lubrication, a shock absorption in the tissue, supporting collagen and elastin in the cellular spaces and keeping protein fibers in balance. GAGs consist of long unbranched polysaccharides containing a repeating disaccharide units with the primary configurations containing either of two modified sugars, *N*-acetylgalactosamine (GalNAc) or *N*-acetylglucosamine (GlcNAc), and a uronic acid such as glucuronate (GlcA) or iduronate (IdoA)^13,14^. Abnormal concentrations of glycosaminoglycans (GAG) have been reported for various types of tumors, suggesting that they may play a role in neoplasia^2,9^. Sulfated glycosaminoglycans (Chondroitin sulfate) were reported to play a major role in breast cancer metastasis^10^.

It has also reported that GAGs play a very important role in brain during development^15^. On the basis of their results the authors proposed that the higher amounts of hyaluronic acid found in very young brain may be responsible for the higher water content of brain at these ages, and that the hydrated hyaluronic acid serves as a matrix through which neuronal migration and differentiation may take place during early brain development^15^. The role of glycan-binding proteins (GBPs) in brain on neuronal cells and neurite outgrowth has been highlighted^16^.

Given the structural diversity of glycans, major barrier in development of glycomics until recently have been the lack of proper tools. Recent technological advances in capillary electrophoresis (CE) and high-performance liquid chromatography (HPLC) separation methods, laser-induced fluorescent detection (LIF), high pH anion-exchange chromatography (HPAEC) with pulsed–amperometric detection, matrix-assisted laser desorption ionization (MALDI) and electrospray mass spectrometry have significantly lowered these barriers^17–20^. Identifying the structure, and function of glycans in cellular biology is a daunting task that has catalysed the emergence of a new field called glycomics^21^. Numerous methods have emerged for decoding the glycome. In the last decade, glycan microarrays have revolutionized the analysis of the specificity of glycan-binding proteins (GBPs), providing information that simultaneously illuminates the biology mediated by them and address the diversity of the human glycome^22^.

Raman spectroscopy can revolutionize the ‘omics’ research largely due to their analytical power. The statement ‘omics research’ refers to a field of biological sciences that ends with *-*omics, such as genomics, transcriptomics, proteomics, lipidomics or metabolomics.

Raman-driven approach provides the opportunity to extend the traditional methods of conventional biology to determine biomarkers by their unique vibrational signatures. New Raman methods (Raman imaging, near field scanning optical microscopy (SNOM), tip enhanced Raman spectroscopy (TERS), surface enhanced Raman spectroscopy (SERS)) have been widely used in biomedicine and greatly accelerated advances in the field^23–28^. Goodacre and Deckert showed for the first time that TERS can be used to distinguish successfully between glycosylated and nonglycosylated proteins from the measurements of just a few molecules within a monolayer^28^.

The goal of this paper is to examine whether Raman spectroscopy and Raman imaging are useful to identify glycoforms of glycoproteins in a cell or tissue and to map glycan attachment sites in normal and cancerous tissues.

## Results and Discussion

Here, we will examine if Raman spectroscopy and Raman imaging are useful to define all of the molecular species, particularly glycoforms of glycoproteins, in a cell or tissue. The biochemical compositional change of cells and tissues associated with abnormality of cancer can aid our understanding of metabolic pathways and mechanisms of cancer development. In contrast to conventional methods used in ‘omics’ field Raman imaging offer both molecular specificity and spatial resolution utilizing the Raman scattering of electromagnetic radiation of the sample constituents to reveal chemical composition and distribution in the tissue. Here, we will show the characteristic vibrational Raman images and Raman spectra in the fingerprint and high frequency spectral regions of the cancer tissues in different regions including lipid-rich, protein-rich, glycan-rich regions of tumors in human breast and brain cancers. To draw greater attention to the opportunities afforded by Raman spectroscopy glycan attachment sites will be mapped, which is a prerequisite for understanding their functions.

**Figure 1:**
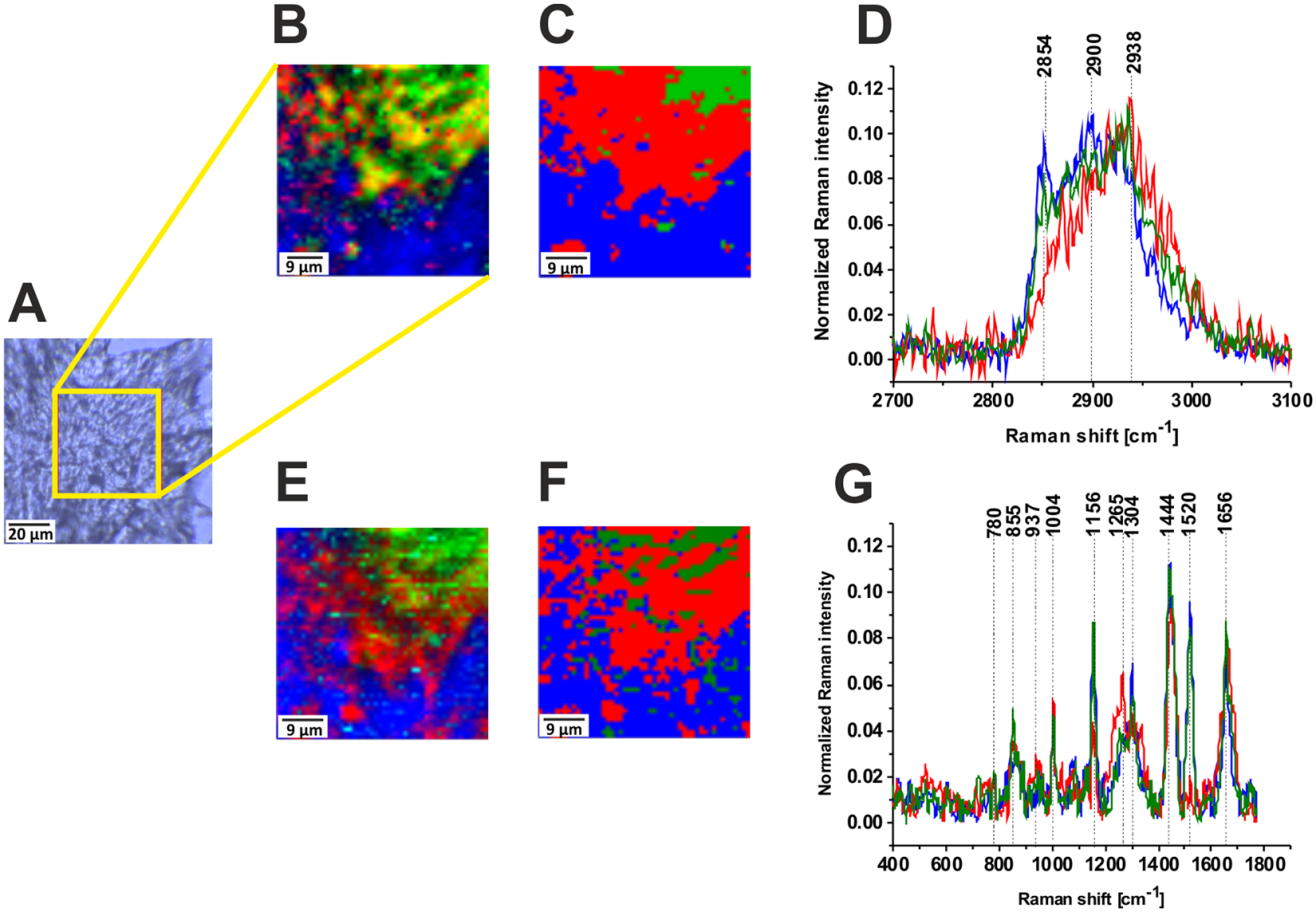
Distribution of the proteins (red), lipids-carotenoids (green) and lipids (blue) in the human breast tumor tissue, the white light microscopy image (**A**), Raman image (50 × 50 µm) obtained from the basis analysis (**B**), Raman image obtained from the cluster analysis for 3 clusters (**C**) and Raman spectra (**D**) in the high frequency spectral region. Raman image obtained from the basis analysis (**E**), Raman image obtained from the cluster analysis for 3 clusters (**F**) and Raman spectra (**G**) in the fingerprint spectral region of the cancer breast tissue (Patient P157, adenocarcinoma G1), integration time for Raman images 0.5 s in the high frequency region and 1 s in the low frequency region, resolution step 1 µm, laser excitation power 10 mW. The line colors of the spectra correspond to the colors of the Raman maps.

Figure 1 shows distribution of the main constituents of the cancer human breast tissue in Raman images. One can see that they are dominated by proteins (red), lipids-carotenoids (green) and lipids (blue). The blue region corresponds to that part of the section free of cancer cells while the red region corresponds to the tumor mass.

Figure 1D and G show also the characteristic vibrational Raman spectra corresponding to the specific structures identified by the Raman images. The Raman profiles free of cancer cells are dominated by lipid-carotenoid-rich region (blue and green colors). The Raman profiles where cancer cells have been found are dominated by protein-rich regions (red color) in the fingerprint and high frequency spectral regions of human breast tumor tissue (Elston-Ellis (WHO) G1, adenocarcinoma).

Knowing from Fig. 1 that cancer cells are heterogeneously distributed in the cancer breast tissue from the tumor mass and contains lipid-carotenoid- rich and protein-rich regions, it is valuable to compare this distribution with that in the normal tissue from the “margins of resection,” referring to the surrounding tissue that is removed along with a tumor.

**Figure 2:**
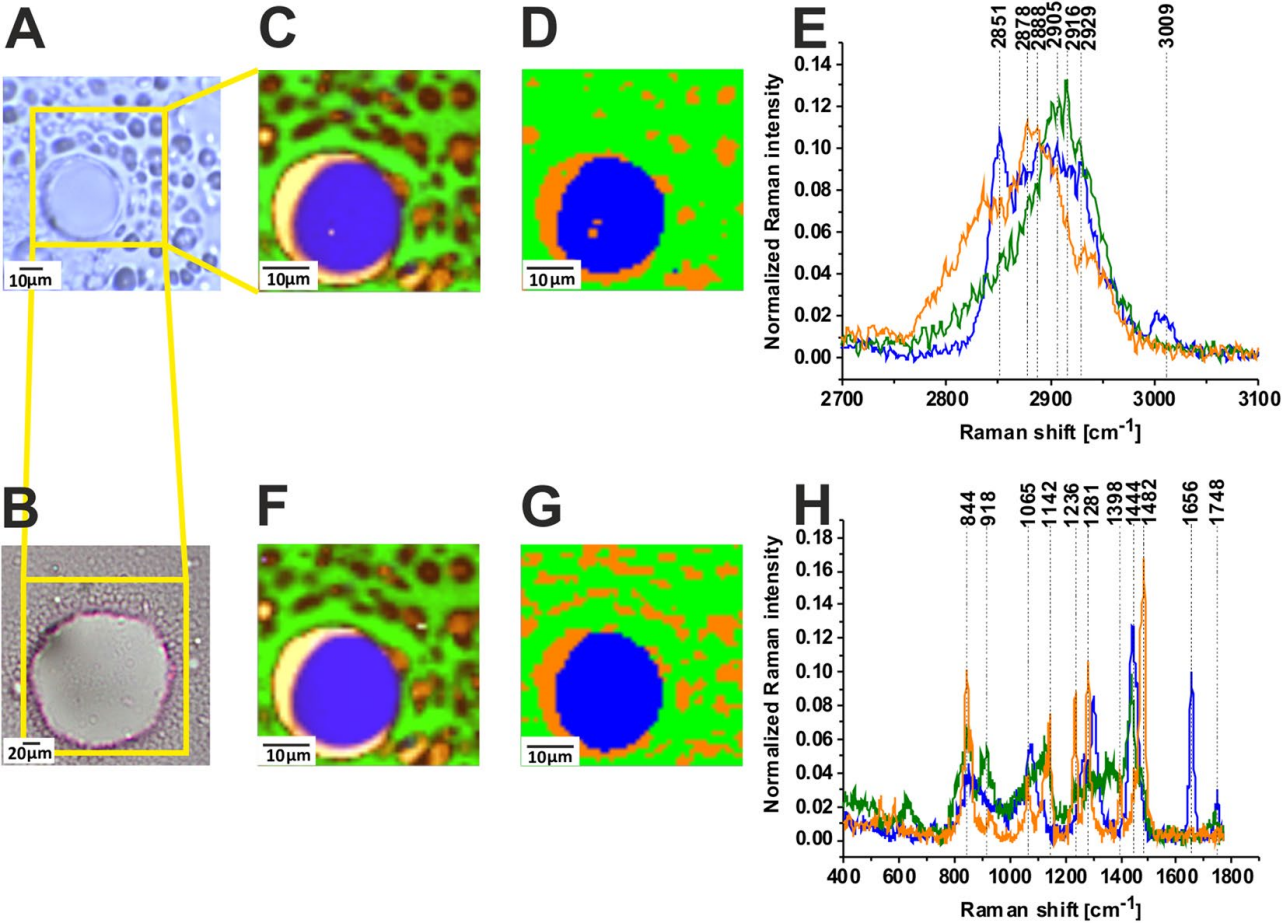
Distribution of the glycans (green and orange) and lipids (blue) in the human breast normal tissue, the white light microscopy image (**A**), histopathological image (**B**), Raman image (35 × 35 µm) obtained from the basis analysis (**C**) Raman image obtained from the cluster analysis for 3 clusters (**D**) and Raman spectra (**E**) in the high frequency spectral region. Raman image obtained from the basis analysis (**F**), Raman image obtained from the cluster analysis for 3 clusters (**G**) and Raman spectra (**H**) in the fingerprint spectral region in the human breast normal tissue (Patient P157), integration time for Raman images 0.5 s in the high frequency region and 1 s in the low frequency region, resolution step 1 µm, laser excitation power 10 mW. The line colors of the spectra correspond to the colors of the Raman maps.

Figure 2 shows the distribution of biochemical species in a negative margin where no cancer cells are found from the same patient as presented in Fig. 1. Figure 2E and H show also the characteristic vibrational Raman spectra corresponding to the specific structures identified by the Raman images.

One can see that the structure and biochemical composition of the normal tissue from the negative margin (no cancer cells) of the tumor mass differs spectacularly from the tissue of the tumor mass. Indeed, the Raman images and spectra in the region free of cancer cells in Fig. 2 are dominated by lipid-rich region (blue color) corresponding to adipose tissue which is the main component of the extracellular matrix surrounding the breast tumor mass. Besides of adipose abundant regions the structures of the negative margin are also dominated by glycan-rich regions (green and orange color) clearly visible both in the fingerprint and high frequency spectral regions of human breast normal tissue.

It is worth emphasizing that both methods (basis analysis and cluster analysis) provide identical distribution of the main biochemical constituents of the tissue. Indeed, Fig. 2 shows that the Raman images both in the high (Fig. 2C,D) and low frequency (Fig. 2F,G) spectral regions give identical distribution of biochemical components of the normal breast tissue. Comparison of the clustering results (Fig. 2D and G) with the basis analysis (Fig. 2C and F) shows that we were able to develop a proper tool for biomedical imaging to get an excellent reproducibility of Raman images.

Comparing the spectra from Figs 1 and 2 one can notice spectacular differences between the cancer tissue in Fig. 1 and the normal tissue from the negative margin free of cancer cells in Fig. 2. First, in contrast to the normal breast tissue the cancerous tissue does not express the presence of glycan-rich regions. Second, the protein-rich regions dominate the structure of the tumor mass in contrast to the normal tissue, which is abundant in adipose tissue.

To quantitatively evaluate Raman imaging as a diagnostic tool for identifying processes involved in tumor infiltration we must understand differences in vibrational signatures of Raman spectra among specific cell or tissue regions. The detailed analysis of the Raman spectra and the vibrational assignment are given in Table 1.

**Table 1:** Tentative assignments of the vibrational of the human breast and brain tissue from the Raman spectra.

Wavenumber/cm^−1^	Tentative assignments
582	Glycans
606	Undefined
720	Phospholipid (choline)^1,44,45^
751	Nucleic acids, Trp
825	Lactic acid
840	Tyr, proline, glycogen^46^
858	Glycans, N-acetyloglucosamine, O-S-O (GAG), glycogen
883	Tyr, Lipids/Carbohydrates/Collagen^47^ C-C-N^+^, C-O-C ring, C-C
895	Glycans
917	C-C stretching of proline, glucose, lactic acid^46^
925	Glycans, glycogen, N-acetyloglucosamine
958	Hydroxyproline/Collagen backbone^41,48^ CH=CH bending
997	C-C symmetric stretching, glucose-I-phosphate, symmetric breathing mode of phenylalanine^46^
1004 (R)	Phenylalanine^42,49–51^ symmetric ring breathing of protein^51^
1064	Lipids/Collagen^41,47^ C-C str.
1091	Phospholipids, O-P-O symmetric stretching^41^, P=O symmetric vibration from nucleic acids/cell membrane phospholipids
1120	GAG, S=O stretching
1080–1158	Proteins (C-C/C-N str.)^47,52,53^, P=O symmetric vibration from nucleic acids and phospholipids
	L-Tryptophan^51^
1189 (R)	C-C_6_H_5_ Phe, Trp^47^
1238	Phospholipid, O-P-O antisymmetric stretching^54^ Amide III β−sheet^46^
1242	GAG S=O stretching
1248	Nucleic acids (Try, Ala)/Proteins (Amide III β sheet or random coil), Lipid, phospholipid =C-H bending^41,47^
1267	Fatty acids, =C-H bend^41,47^
1276	Amide III^46^ α helix, P=O asymmetric stretching due to nucleic acids
1281	GAG
1304(R)	Lipids, phospholipids^41^ C-H_2_ twist, collagen, protein amide III^46^, DNA^46^
1327	N-acetyloglucosamine
1339/1370	Trp, C_a_-H def
1378	N-acetyloglucosamine
1437–1444 1453	Fatty acids, triglycerides, CH_2_ or CH_3_ deformations^53^ Proteins^3,51^ C-H wag, CH_2_ or CH_3_ def. Phospholipids, CH_2_ scissoring^55^
1484	Glycans
1558	Amide II, proteins^41,48^, amide II β−sheet^52^
1584	Amide II^45^, aromatic amino acids within proteins^53^, nucleic acids^41,47^
1651	(C=C) stretching, unsaturated fatty acids, triglycerides
1658	(C=C) stretching^53^, Amide I α helix
1667–1680	Proteins, Amide I β−sheet, cholesterol esters^46^
1667–1680	Proteins Amide I turn^44^/Unsaturated fatty acids^47,51^, (C=O) stretching, (C-H) def./(C=C) str.^47,51^, collagen, elastin^41^
1745 1731	(C=O) stretching, triglycerides (C=O) stretching, glycans, glycogen
2845/2854	Fatty acids, triglycerides, C-H_2_ symmetric stretching
2888	Lipids^51^, C-H_2_ antisymmetric stretching
2905	proteins/lipids C-H_2_ antisymmetric stretching^56–59^ CH_3_ symmetric stretching^41,58,60^ C-H_2_ antisymmetric stretching, Lysine^23^, glycogen
2931/2940	Proteins/Lipids, CH_3_ symmetric stretching^41,51^ CH_3_ symmetric stretching Acetylated lysine^23^ Proteoglycans, Heparan sulfate, Chondroitin sulfate
2963	Methylated lysine^23^
3009	Lipids^41,51^=C-H stretching
3067	Nucleic acids/Proteins^51^ C-H aromatic

**Figure 3:**
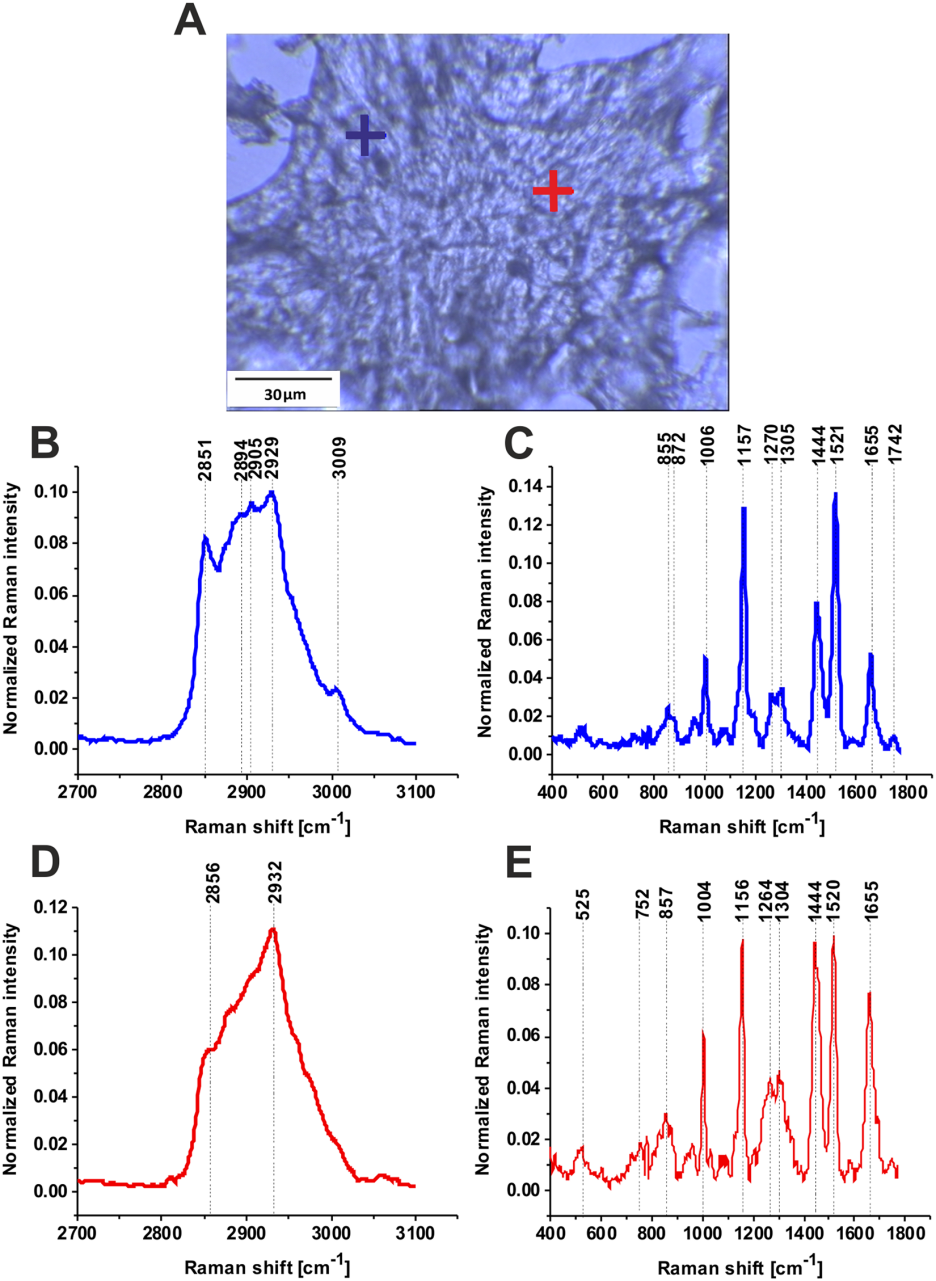
Typical Raman profiles in cancerous breast tissue. Microscopy image 145 µm × 110 µm (**A**), and characteristic vibrational Raman spectra in the lipid-rich region (blue cross) and the protein-rich region (red cross) in the white light microscopy image) (**A**) in the high frequency spectral region (**B**,**D**) and low frequency region (**C**,**E**) of the human breast tumor tissue (Patient P157, adenocarcinoma G1, Elston-Ellis (WHO) G1), integration time for a single spectrum 2 s, 10 accumulations, laser power 10 mW, P157. The color of crosses corresponds to the colors of the Raman spectra P157.

Figure 3 shows the characteristic vibrational Raman profiles in cancerous breast tissue from selected points of the tissue. The typical Raman profiles are dominated by lipid-carotenoid-rich region (blue cross) and protein-rich regions (red cross) in the fingerprint and high frequency spectral regions of human breast tumor tissue (Elston-Ellis (WHO) G1).

The lipid-carotenoid-rich region is characterized by the Raman peaks: 2851, 2894, 2905, 2929 cm^−1^ (Proteins/Lipids, CH_2_ and CH_3_ stretching), 1742 cm^−1^ (lipids, C=O stretching, triglycerides), 1655 cm^−1^ (C=C stretching of unsaturated lipids), 1521 cm^−1^ (carotenoids), 1437 cm^−1^ (lipids, CH_2_ and CH_3_ deformations), 1304 cm^−1^ (lipids, CH_2_ twist), 1270 cm^−1^ (lipids, =C-C bending), 1157 cm^−1^ (carotenoids), 1063 cm^−1^ (phospholipids), 872 cm^−1^ (lipids, C-C deformations), 720 cm^−1^ (choline). Most of these peaks have been reported as the most reliable candidates for Raman biomarkers and therapeutic of normal breast tissue^24–27,29^.

**Figure 4:**
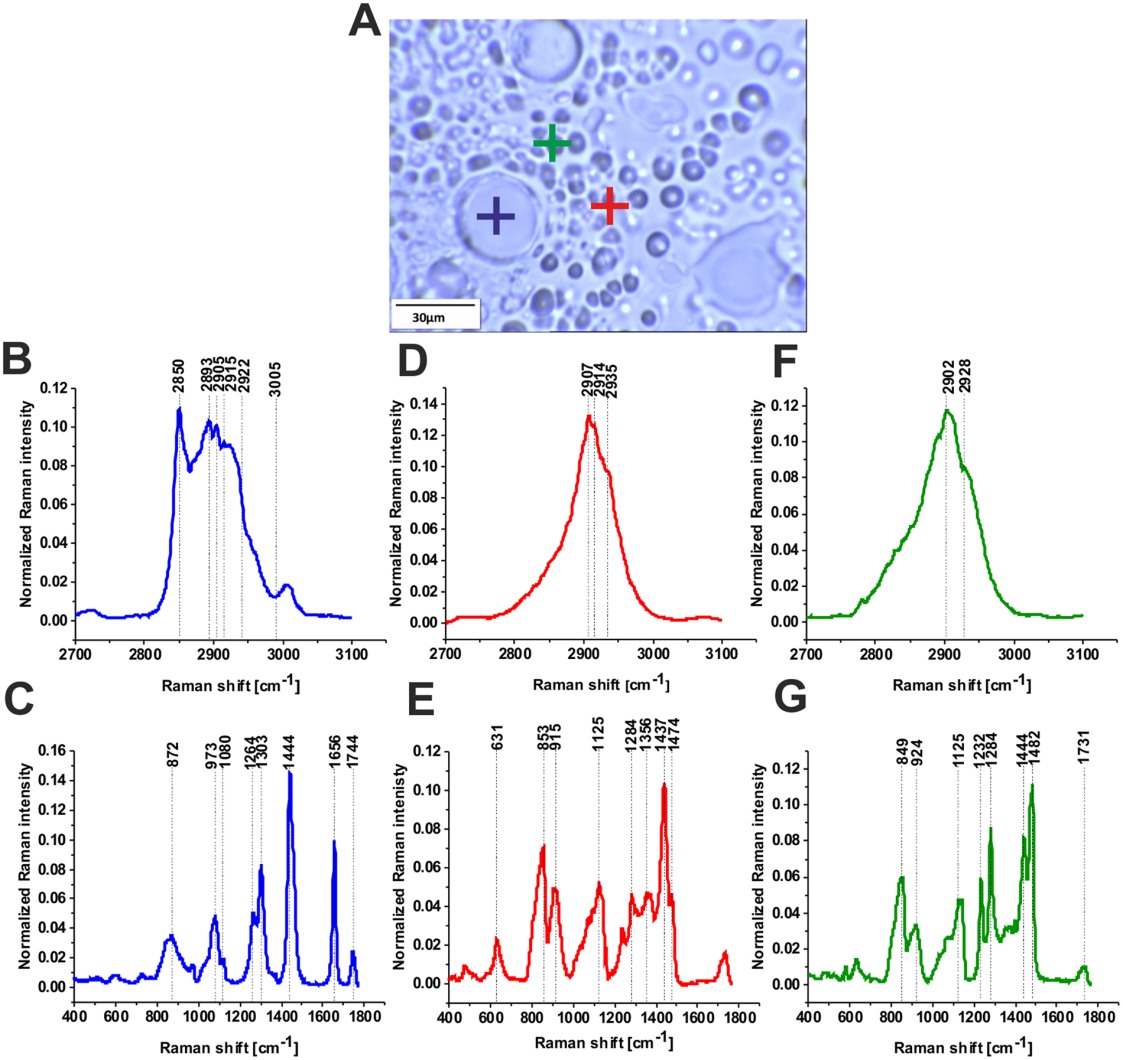
Typical vibrational Raman spectra in normal breast tissue from the tumor margin (for the same patient P157 as in Fig. 3), lipid-rich region (blue cross) and glycan-rich regions (red and green cross) in the white light microscopy image (**A**)) in the high frequency (**B**,**D**,**F**) and in the fingerprint (**C**,**E**,**G**) spectral regions of human breast normal tissue, integration time for a single spectrum 2 s, 10 accumulations, laser power 10 mW, patient P157.

Figure 4 shows the typical vibrational Raman spectra in the normal breast tissue from the tumor margin (for the same patient P157). One can see that the Raman profiles are dominated by lipid-rich region (blue cross) and glycan-rich regions (green cross) in the fingerprint and high frequency spectral regions of human breast normal tissue. Detailed inspection in the Figs 3 and 4 demonstrate that the most spectacular difference between the cancerous and normal breast tissue of the same patient (P157) is related to glycan-rich regions. Indeed, the normal breast tissue is heavily abundant in glycans in contrast to the cancerous tissue that is dominated by proteins.

In the view of the results presented so far we will concentrate on the glycan-rich regions. It is perhaps no wonder that this issue attracts our attention, because many tumor-specific antigens have been discovered to be cell surface carbohydrate structures^30^. Clinical cancer diagnostic markers very often belongs to glycoproteins family. The National Cancer Institute has begun an initiative to discover, develop, and clinically validate glycan biomarkers for cancer^31^.

The Raman glycan-rich Raman profiles, absent in the cancerous tissue (Figs 1 and 3), are clearly visible in the normal breast tissue (green color spectra in Figs 2 and 4). Glycans are often profiled after their release from polypeptides, which results in the loss of any information about proteins and sites to which they were attached^2,11^. It is obvious that the glycans presented in Fig. 4 do not represent proteoglycans (heavily glycosylated proteins covalently attached GaG chains), because the spectra lack any vibrations typical for proteins (e.g. 1656 cm^−1^). Detailed inspection in Fig. 2 shows that one can distinguish two types of glycan structures (orange and green color in Fig. 2). The main difference between the structure is the Raman band at 1482 cm^−1^. The Raman green glycan-rich profiles (glycoform GI) in Fig. 2 is dominated by the peaks at 627 cm^−1^, 844 cm^−1^, 918 cm^−1^, 1120 cm^−1^, 1354 cm^−1^, 1438 cm^−1^, 1734 cm^−1^. The Raman orange glycan-rich profiles (glycoform G II) in Fig. 2 is dominated by the peaks at 532 cm^−1^, 587 cm^−1^, 844 cm^−1^, 930 cm^−1^, 1065 cm^−1^, 1142 cm^−1^, 1236 cm^−1^, 1281 cm^−1^, 1482 cm^−1^.

To identify the glycan structures corresponding to the Raman profile we have compared the glycan-rich profile with the pure components of N-acetylo glucosamine (an amino sugar, a monosaccharide and a derivative of glucose), glycogen-a polysaccharide that is the main form of carbohydrate storage in animals, glycosaminoglycans (GAGs) which are long unbranched polysaccharides consisting of a repeating disaccharide unit (heparan sulfate, chondroitin A, chondroitin B, hyaluronic acid), and proteoglycans that are heavily glycosylated proteins (heparan proteoglycan, proteoglycan). Chondroitin sulfate and heparan sulfate are highly sulfated GAGs in contrast to hyaluronic acid which is not sulfated.

**Figure 5:**
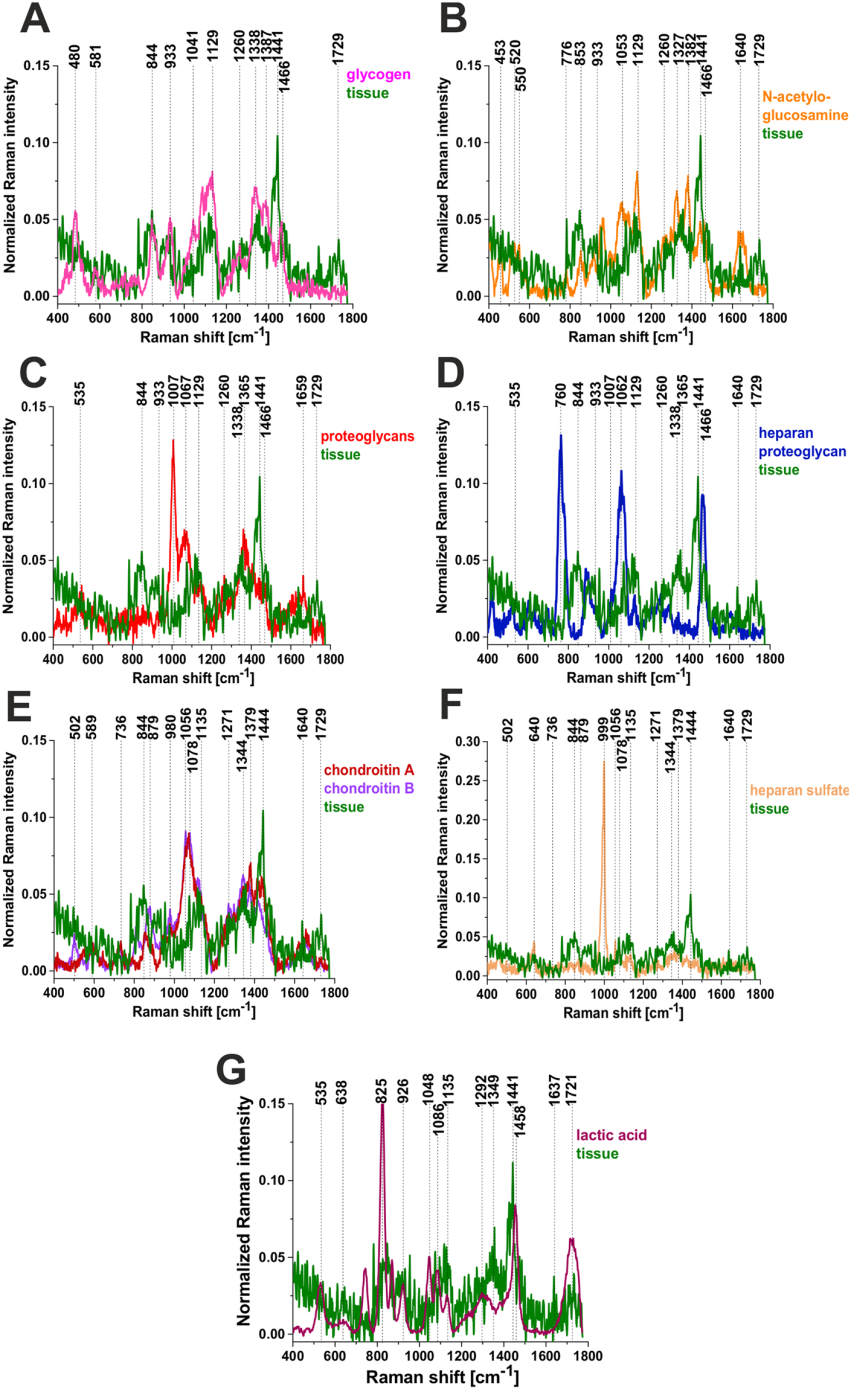
The comparison of the typical glycan-rich Raman spectrum with glycogen (**A**) N-acetylo-glucosamine (**B**), proteoglycans (**C**), heparan proteoglycan (**D**), chondroitin A and B (**E**), Heparan sulfate (**F**), lactic acid (**G**).

Figure 5 shows the comparison of the typical glycan-rich Raman spectrum with glycogen, lactic acid, the representative of sulfonated and non-sulfonated GAG’s and proteoglycans. The best reproduction of the tissue experimental data for glycoform I is obtained for glycogen and N-acetyloglucosamine in the low frequency region, and for glycogen in the high frequency region. We have assigned the vibrations (844 cm^−1^, 918 cm^−1^, 930 cm^−1^, 1065 cm^−1^, 1120 cm^−1^, 1142 cm^−1^) to C-O-C and C-OH of polysaccharides. A peak at 1482 cm^−1^ is a characteristic feature of CH_3_ symmetric deformation vibrations observed in standard GAGs. Some of these peaks may overlap with less intense sulfate vibrational modes observed at 853 cm^−1^ (OSO GAG), 1125 cm^−1^ (S=O GAG) and 1232 cm^−1^ (S=O GAG). The peak at 1734 cm^−1^ shifted significantly from the peak of C=O stretching vibration of triglycerides have been assigned to C=O vibration of sulfated GAGs (chondroitin sulfate). The glycoform GII of glycan-rich region (orange color in the Raman spectra and Raman images) is more difficult for reliable interpretation. Preliminary the strong band at 1482 cm^−1^ have been assigned to heparan proteoglycans. The peaks at 2835 cm^−1^ and 2878 cm^−1^ has been assigned to N-acetylo glucosamine. Some of the assignments correlate with those of ref.^32^. It is worth emphasizing that hyaluronic acid, although distributed widely throughout connective, epithelial and neural tissues and is one of the chief components of the extracellular matrix, which contributes significantly to cell proliferation and migration, and progression of some malignant tumors^33^, does not reproduce well the Raman features observed in the glycan-rich region (profile GI).

**Figure 6:**
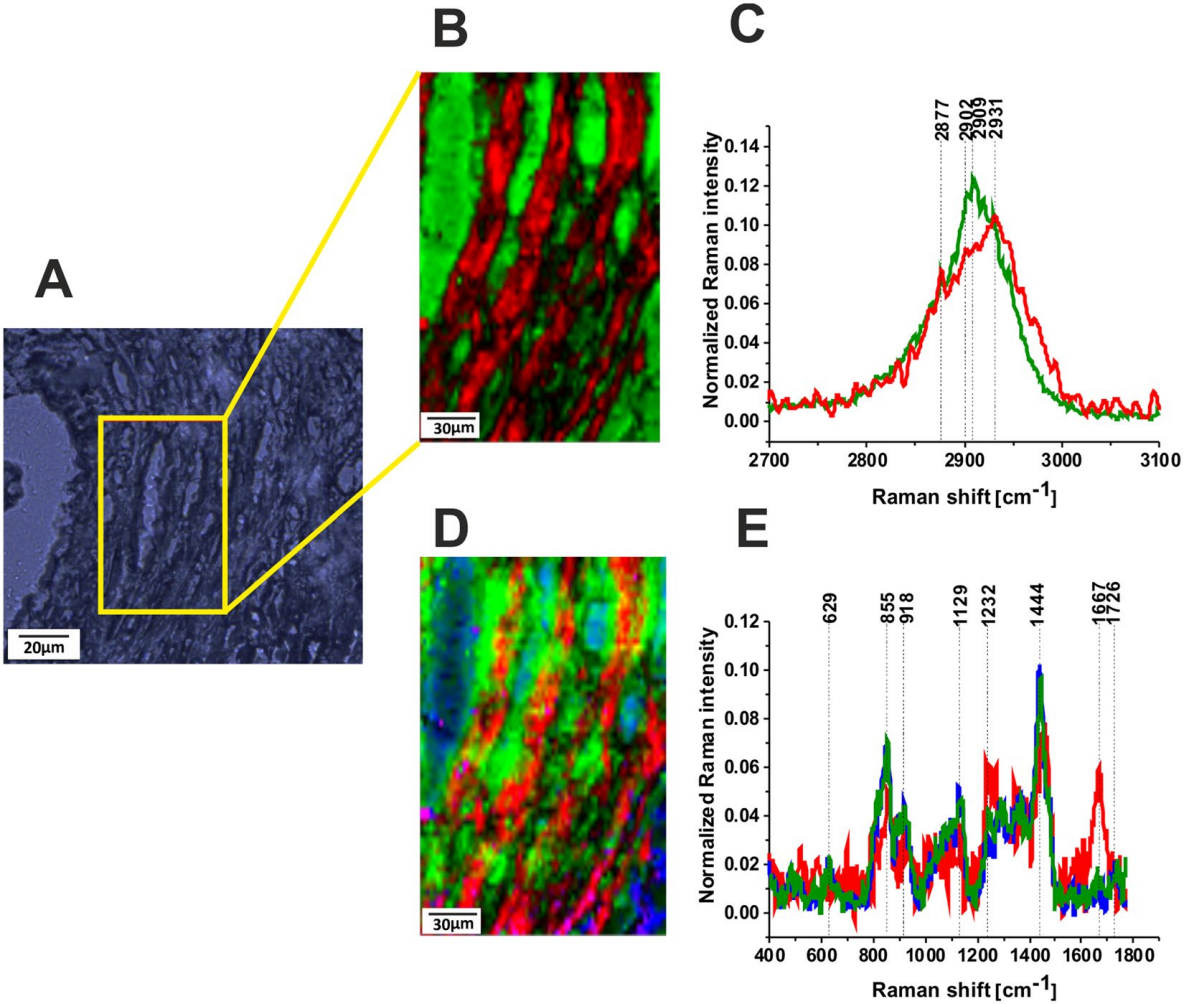
Distribution of the glycans (green), lipids (blue) and protein (red) in the human breast tumor tissue, the white light microscopy image (**A**), Raman image (150 µm × 230 µm) obtained from the basis analysis (**B**) and Raman spectra (**C**) in the high frequency spectral region. Raman image obtained from the basis analysis (**D**) and Raman spectra (**E**) in the fingerprint region of the tumor breast tissue (Patient P155, Infiltrating adenocarcinoma grade WHO according to Elston and Ellis modification G2), integration time for Raman images 0.5 s in the high frequency region and 1 s in the low frequency region, resolution step 0.5 µm, laser excitation power 10 mW. The line colors of the spectra correspond to the colors of the Raman maps.

Although the results presented so far for some patients (see Figs 1 and 2) may suggest that the normal breast tissue is much more heavily glycolisated than the cancerous tissue, our results for another group of patients demonstrates that the glycoform GI is not specific only for normal tissue, but has also been found in the cancer tissue. Figure 6 shows the distribution of biochemical species in the cancerous breast tissue. Figure 6 shows that the glycan-rich regions (green color) are also present in the cancerous breast tissue.

The Raman maps in Fig. 6D clearly demonstrate that glycans are predominantly attached to lipids (overlapping of blue and green areas).

**Figure 7:**
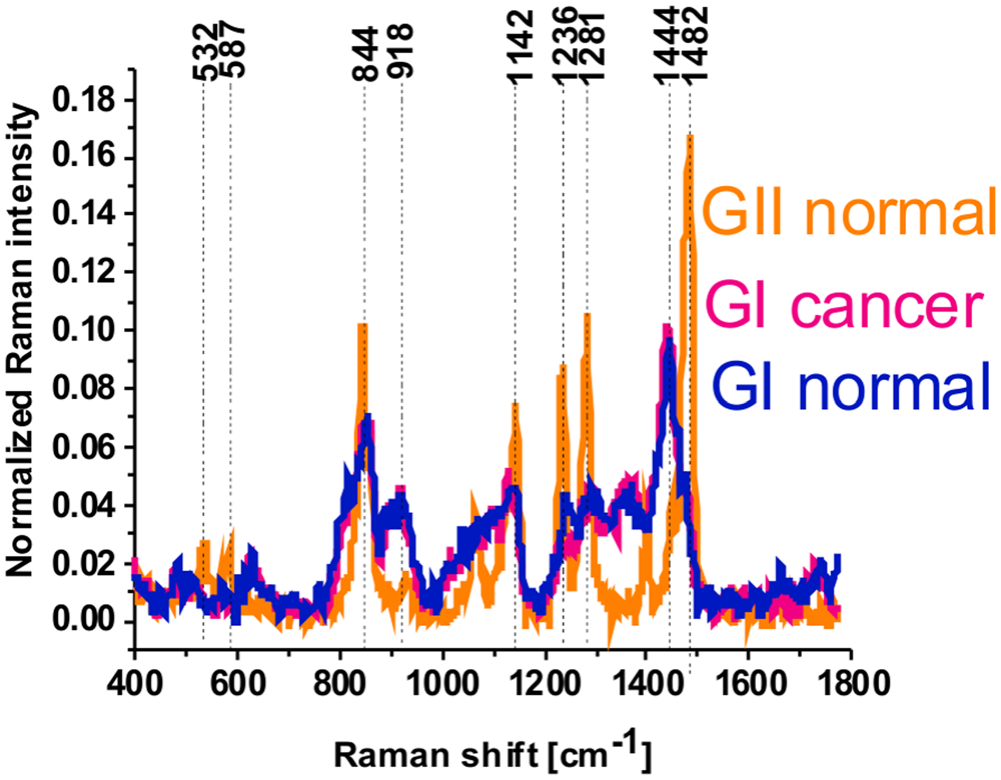
Raman glycoform of the cancerous breast tissue (P155) (GI cancer) compared with the GI and GII glycoforms (GI normal, GII normal) of the normal breast tissue (P157).

The Raman glycoform of the cancerous breast tissue from Fig. 6 has been compared with the GI and GII glycoforms of the normal breast tissue from Fig. 2 and they are presented in Fig. 7.

One can see that the glycoforms GI of the normal and cancerous breast tissues are identical, but the glycoform GII is specific for the normal breast tissue and it has not been found in the cancer tissue.

**Figure 8:**
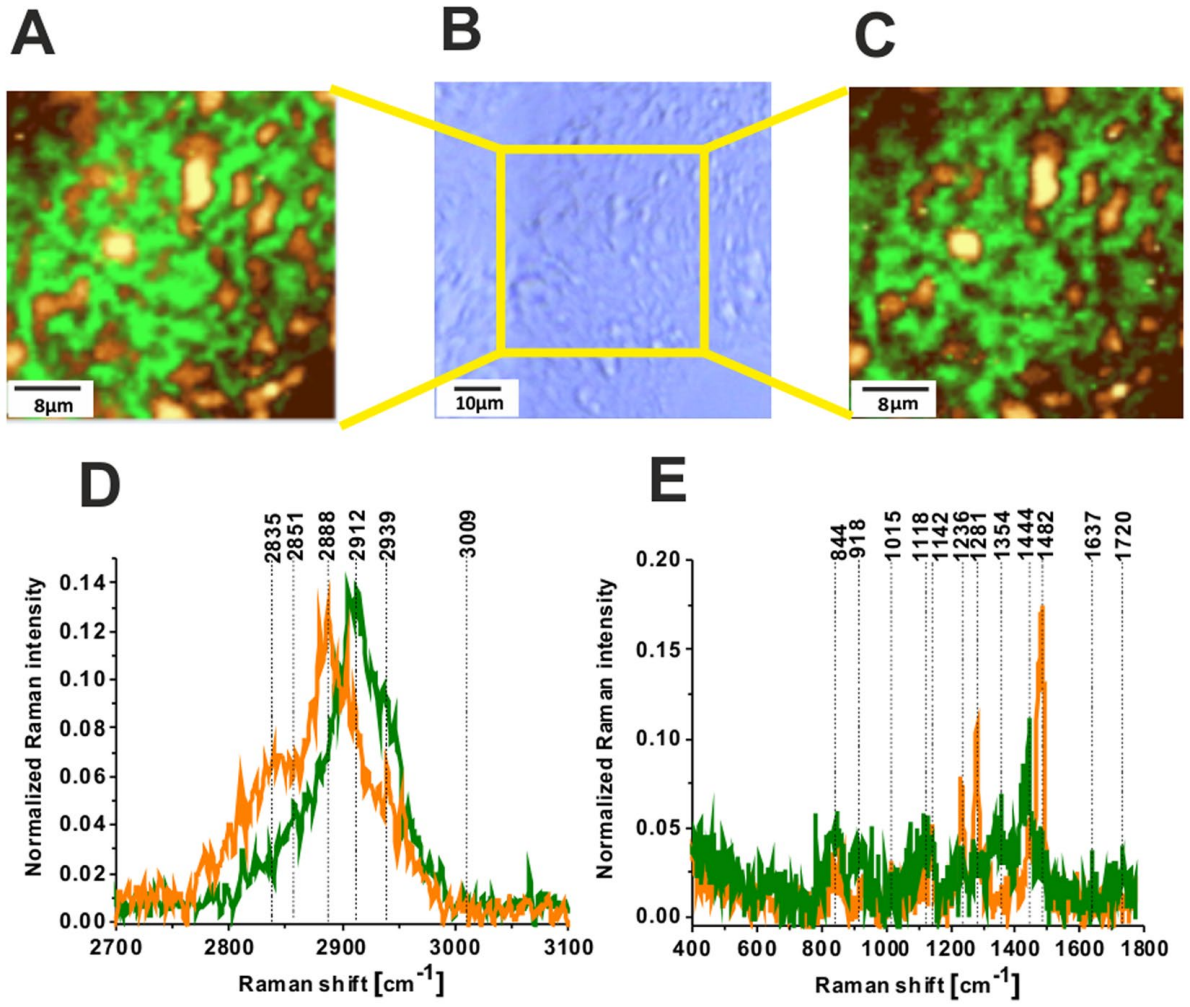
Raman image of glycans for brain tumorous tissue (P2, Medulloblastoma, WHO IV), (40 × 40 µm) in high frequency region (**A**), the white light microscopy image (65 × 65 µm) (**B**), Raman image (40 × 40 µm) in fingerprint region (**C**), Raman spectra in high frequency (**D**) and fingerprint spectral region (**E**) for brain tumorous tissue (P2, Medulloblastoma, WHO IV), integration time 0.5 s, resolution step 0.5 µm, laser excitation power: 10 mW. The line colors of the spectra correspond to the colors of the Raman maps.

It is interesting to learn if the glycoform Raman profiles are typical only for breast cancer or represents a more universal hallmark for other types of cancer. Figure 8 shows Raman images and Raman spectra for brain tumorous tissue (P2, Medulloblastoma, WHO IV).

The orange and green regions of Raman images in Fig. 8A,C correspond to the Raman spectra in Fig. 8D,E in the tumorous brain tissue. The presented results show that both glycome Raman profiles GI and GII of glycan structures exist in brain tumorous tissue. The standardization will be necessary to measure overexpression or underexpression of glycans, which is a prerequisite for understanding their functions. The results obtained by Raman approach for the role of glycans in cancer support the results obtained by the methods of conventional biology and they can be a valuable alternative^34–40^. Raman-driven approach provides the opportunity to extend the traditional methods of conventional biology to determine biomarkers by their unique vibrational signatures.

## Conclusions

Direct visualization of the epithelial glycocalyx are crucial to establish its exact role in mechanisms of cancer development. So far the staining of glycocalyx by specific markers made them detectable by fluorescence and TEM. We have shown that Raman microspectroscopy is a powerful method for visualization of glycocalyx offering cellular interrogation without staining, unprecedented spatial and spectral resolution to provide important biochemical information. High spatial resolution of Raman imaging combined with chemometric procedures allows separation of individual species from many chemical components present in each cell. We have shown that the Raman imaging is capable of recording vibrational spectra from multiple regions and thereby map out the spatial distribution of proteins, lipids, nucleic acids, and glycans in human tissue in contrast to the classical methods LC/MS, NMR, HPLC, based on the analysis of samples in the mass subjected to homogenization that prevents spatial characteristics of the systems investigated.

Attachment sites of proteins, lipids, and glycans have been mapped in breast and brain human tissues. Our results demonstrate that Raman imaging is a sensitive and specific method to identify structures and map attachment sites and distribution of glycans in markedly heterogeneous tissue. Raman markers characteristic of glycogen, glycosaminoglycan, chondroitin sulfate, heparan sulfate proteoglycan were identified based on their vibrational signatures. Raman spectral signatures of glycosaminoglycans (GAGs) in human breast and brain tissues were demonstrated. Raman microspectroscopy allowed probing glycosaminoglycan distribution in the normal and cancerous tissues. We have found that metabolism of proteins, lipids and glycans is markedly deregulated in breast (adenomacarcinoma) and brain (malignant medulloblastomas) cancers. We have identified two glycoforms GI and GII in the normal breast tissue and the malignant brain tissue in contrast to the breast cancer where only one glycoform GI has been identified.

Given that these changes in glycan structures occur as a result of oncogenic transformation and have impact on proteins and lipids functions Raman imaging may cause a significant progress in the diagnosis and treatment of cancer by providing information about the spatial location of the glycans binded to protein and lipids in tissues and cells. The novel methods of Raman imaging combined with the conventional tools of genomics, proteomics, lipidomics, and metabolomics can make significant progress in glycomics studies. The novel approach will help to monitor changes resulting from the altered expression of glycosyltransferases by the transformed cells where overglycosylation are normally due to elevated expression or induction of specific glycosyltransferases and underglycosylation is often due to repression of specific glycosyltransferases. The standardization of Raman method will be necessary to measure overexpression or underexpression of glycans, which is a prerequisite for understanding their functions.

## Methods

### Chemicals

The following chemicals have been purchased from Sigma-Aldrich: Proteoglycan from bovine nasal septum (P5864; Sigma Aldrich), Heparan sulfate sodium salt from bovine kidney (H7640; Sigma Aldrich); Heparan sulfate proteoglycan (H4777; Sigma Aldrich), Chondroitin sulfate B sodium salt (C3788; Sigma Aldrich), Chondroitin sulfate A sodium salt from bovine trachea (C9819; Sigma Aldrich), N-Acetyl-D-glucosamine (A8625; Sigma Aldrich).

### Tissue preparation for Raman measurements

All experiments were performed in accordance with relevant guidelines and regulations of the Bioethical Committee at the Polish Mother’s Memorial Hospital Research Institute in Lodz (53/216), and by the institutional Bioethical Committee at the Medical University of Lodz, Poland (RNN/323/17/KE/17/10/2017). Written informed consent was obtained from all patients, or if subjects are under 18, from a parent and/or legal guardian.

The brain tissue sections were obtained at the Polish Mother’s Memorial Hospital (Lodz, Poland). The breast tissue sections were obtained at the Voivodship Nicolaus Copernicus Specialist Hospital in Lodz. The 16 µm thick slices of brain and breast sections were put on CaF_2_ windows. For reference, brain and breast tissues were stained with H&E staining method. Histology examination for all the specimens was performed by certified neuropathologist and histopathologists from the Polish Mother’s Memorial Hospital Research Institute and the Medical University of Lodz. For spectroscopy analysis we used only fresh tissue, because formalin-fixed paraffin-embedded (FFPP) tissues alter phenotype. Detailed description of methodology is available elsewhere^1,2,29^.

### Study participants

All research in the study were approved by Bioethical Committee at the Polish Mother’s Memorial Hospital Research Institute in Lodz (no. 53/216), and by the institutional Bioethical Committee at the Medical University of Lodz, Poland (no. RNN/323/17/KE/17/10/2017). Written informed consents were obtained from all patients. The tissue samples consisted of medulloblastoma (grade IV) (n = 5) and breast cancer (n = 7) (Infiltrating adenocarcinoma grade II, adenocarcinoma grade I). As controls we used the tissue from the negative margins.

### Raman spectroscopy and Raman imaging

All Raman spectra and Raman imaging were recorded with confocal Raman microscope – WITec alpha 300 RSA+ (Ulm, Germany) equipped with Olympus microscope, coupled with monochromator (Princeton Instruments Acton SP23000-300 mm Imaging Triple Grating Monochromator/Spectrograph) and CCD Camera ANDOR Newton DU970N-UVB-353 (EMCCD chip with 1600 × 200 pixel format, 16 µm dimension each) operating in the standard mode at −64 °C with full vertical binning. Samples were excited by laser operating at 532 nm. For Raman spectra and Raman maps collection 40x magnification objective (NIKON CFI Plan Fluor C ELWD 40x: NA 0.60, WD 3.6–2.8 mm; DIC-M, C.C.0-2) was used. Detailed description of methodology is available elsewhere^1,23–27^. To analyze all presented results we used diode laser (SHG Nd:YAG, with a matrix (Y3Al5O12) doped with neodymium ions (Nd3+)) operating at 532 nm. The average laser excitation power was 10 mW. The data has been recorded with a collection (integration) time of 0.5 s and a spectral resolution of 2 cm^−1^ in the fingerprint range of 200–1800 cm^−1^ and the high frequency region of 2100–3500 cm^−1^. Every day before Raman measurements the confocal system was calibrated using silicon plate (520.7 cm^−1^).

### Data analysis methods

All collected Raman data were preprocessed using the WITec Project 4.1. Each spectrum was processed to remove cosmic rays, smooth (Savitzky-Golay method) and remove background. To remove Rayleigh scattering that has not been cut off by a filter and the Raman spectrum of CaF_2_ support spectral range has been limited to 400–1800 cm^−1^. The spectra were normalized by vector norm. To prepare Raman color-code images the basis analysis methods (BAM) with Manhattan metrics and K-means cluster analysis (KMCA) methods with Manhattan metrics were applied.

The BAM approach is based on the average spectra from various areas of the sample. In the BAM method each recorded spectrum is compared to the selected average spectra BS_A,_ BS_B,_ BS_c_ by using a least squares fit to minimize the fitting error D described by the equation given below

$$D={([\overrightarrow{Recordedspectrum}]-\alpha x\overrightarrow{B{S}_{A}}-bx\overrightarrow{B{S}_{B}}-cx\overrightarrow{B{S}_{C}}-\ldots )}^{2}$$

where a, b, c, … are the weighting factors of the selected basis spectra BS. The color code of the monochromatic Raman maps corresponds to Raman intensities in the specific regions with the weight factors of each point converted to monochrome intensity map. The bright yellow colors and dark brown colors correspond to the highest intensities and the lowest intensities, respectively. The monochrome maps are converted to colors and combined to yield a pseudo-color Raman map with mixed colors to visualize the distribution of the weight factors representing chemical composition of the sample.

In KMCA methods Raman spectra are sorted according to their similarities. K-means cluster analysis is based on k clusters consisting of n_k_ spectra. Each spectrum belong to the cluster with the nearest mean. The images created by BAM and KMCA have been compared to reach full correspondence and coherence between them. Detailed methodology on pre-processing data analysis used in the paper is available elsewhere^25,41–43^.

## Data Availability

All data generated or analyzed during this study are included in this published article.
